# Effect of *α*″-Ti Martensitic Phase Formation on Plasticity in Ti–Fe–Sn Ultrafine Eutectic Composites

**DOI:** 10.3390/mi15010148

**Published:** 2024-01-19

**Authors:** Deva Prasaad Neelakandan, Wonhyeong Kim, Barton C. Prorok, Elham Mirkoohi, Dong-Joo Kim, Peter K. Liaw, Gian Song, Chanho Lee

**Affiliations:** 1 Department of Materials and Mechanical Engineering, Auburn University, Auburn, AL 36849, USA; dzn0037@auburn.edu (D.P.N.); wzk0024@auburn.edu (W.K.); prorobc@auburn.edu (B.C.P.); ezm0095@auburn.edu (E.M.); dkim@auburn.edu (D.-J.K.); 2 Department of Materials Science and Engineering, The University of Tennessee, Knoxville, TN 37996, USA; pliaw@utk.edu; 3 Division of Advanced Materials Engineering, Kongju National University, Cheonan 31080, Republic of Korea

**Keywords:** titanium alloys, eutectic composites, martensitic phase, plasticity, lamellar matrix

## Abstract

Extensive research has been conducted on Ti–Fe–Sn ultrafine eutectic composites due to their high yield strength, compared to conventional microcrystalline alloys. The unique microstructure of ultrafine eutectic composites, which consists of the ultrafine-grained lamella matrix with the formation of primary dendrites, leads to high strength and desirable plasticity. A lamellar structure is known for its high strength with limited plasticity, owing to its interface-strengthening effect. Thus, extensive efforts have been conducted to induce the lamellar structure and control the volume fraction of primary dendrites to enhance plasticity by tailoring the compositions. In this study, however, it was found that not only the volume fraction of primary dendrites but also the morphology of dendrites constitute key factors in inducing excellent ductility. We selected three compositions of Ti–Fe–Sn ultrafine eutectic composites, considering the distinct volume fractions and morphologies of β -Ti dendrites based on the Ti–Fe–Sn ternary phase diagram. As these compositions approach quasi-peritectic reaction points, the $\alpha^{\prime \prime }$ -Ti martensitic phase forms within the primary β -Ti dendrites due to under-cooling effects. This pre-formation of the $\alpha^{\prime \prime }$ -Ti martensitic phase effectively governs the growth direction of β -Ti dendrites, resulting in the development of round-shaped primary dendrites during the quenching process. These microstructural evolutions of β -Ti dendrites, in turn, lead to an improvement in ductility without a significant compromise in strength. Hence, we propose that fine-tuning the composition to control the primary dendrite morphology can be a highly effective alloy design strategy, enabling the attainment of greater macroscopic plasticity without the typical ductility and strength trade-off.

## 1. Introduction

The unique lamellar structure found in ultrafine eutectic alloys is a key factor contributing to their exceptional strength, setting them apart from coarse-grained alloys [1,2,3,4]. This structural feature is characterized by the arrangement of alternating layers of soft and hard phases. The formation of these distinct soft- and hard-phase layers [5,6] plays a pivotal role in enhancing the yield strength of alloys. The soft layers contribute to ductility, while the hard layers offer resistance against deformation. These features, in turn, create a barrier that impedes the movement of dislocations, corresponding to the interface strengthening effect [7,8,9]. Despite these substantial strengthening effects, eutectic alloys have a notable drawback—they tend to be inherently less ductile. This deficiency in ductility arises primarily from stress concentration points within the microstructure [10]. Stress concentrations occur when there are abrupt changes in material properties or geometries, making it easier for cracks to initiate and propagate. In the context of eutectic alloys, the distinct phases and interfaces can become sites for stress concentration, leading to a reduction in ductility.

The primary challenge in the field of advanced structural materials is to strike a balance between enhancing ductility and preserving high-yield strength [11]. To address this significant challenge, there are extensive efforts to develop innovative methods for designing alloys. This approach involves the deliberate addition of minor elements to eutectic compositions, aiming to tailor the microstructures with the improvement in ductility while retaining or even enhancing strength [12,13,14,15,16,17]. This alloy-design strategy holds promise for addressing the critical need for advanced materials that excel in both strength and ductility, providing new potential metallic materials in various engineering applications. Eutectic composites have been used in applications involving aeronautics, aerospace, and power generation technologies because of their resistance to high temperatures. They have been used in 1700 °C-class gas turbine systems without any thermal and environmental barrier coating [18]. Mg-6Ni-3Cu (at. %) eutectic composites are known for a rapid H_2_ absorption rate with a reduced desorption rate; thus, these are suitable candidates for chemical hydrogen storage and potentially being used in fuel cells in the near future [19]. Since there is an active need for materials with enhanced storage capacity in automotive and power generation facilities, eutectic composites could be the needed breakthrough. In addition to this feature, AlCoCrFeNi_2.1_ eutectic high-entropy alloys (HEAs) have been reported to have excellent mechanical properties (yield strength, fracture strength, and elongation) in cryogenic atmospheres [20]. Thin-film resistors are essential components in microelectronic devices and are produced using the magnetron sputtering technique. The temperature coefficient of resistance (TCR) is a critical requirement while making these thin-film resistors. High-entropy thin-film CoCrFeNiTi_x_ deposited on the Si/SiO_2_ substrate demonstrated significant TCR values in the range of −60 °C up to 130 °C, changing its value from 78 ppm/°C at low temperatures to 6.6 ppm/°C at 130 °C [21].

In general, inducing a solid-solution primary dendrites ( β -Ti in present alloys) in a eutectic matrix by tailoring the chemical compositions is a well-known alloy design strategy to enhance the plasticity of Ti Fe ultrafine eutectic alloys [10,22]. The definition of ultrafine alloys is based on the grain diameter. Based on indirect experimental evidence in Fe alloys, the boundary between ultrafine and traditional grains is between 800 and 1300 nm, which again, is alloy-dependent [23]. Later, it was defined that nanocrystalline metals have grain sizes smaller than 100 nm [24]. Ultrafine crystalline alloys have grain sizes between 100 and 1000 nm. Following these, microcrystalline materials have grain sizes in the range of microns. Hence, many extensive efforts have been conducted to improve the plasticity with the maintenance of the high yield strength, and it is found that the mechanical properties of Ti Fe eutectic alloys strongly depend on the morphology and the volume fraction of the dendrite phase and its correlation with the eutectic matrix [25,26,27]. For example, by increasing the volume fraction of the β -Ti dendrite phase up to 30% in (Ti_70.5_Fe_29.5_)_100−x_Sn_x_ with x = 5, 7 and 9; the yield stress decreased from 2.2 GPa to 1.3 GPa, concurrently improving the plasticity from 1.7% to 15.7% [4].

It is well accepted that four non-equilibrium phases, namely $\alpha^{\prime }$ , $\alpha^{\prime \prime }$ , ω , and the metastable β phase exist in Ti alloys [28,29,30]. The supersaturated solid solution phases $\alpha^{\prime }$ and α ″ are formed by rapid quenching from the β -phase field and are referred to as martensite. In pure Ti, martensite is formed in the body-centered cubic (BCC) β -phase field upon quenching. The addition of elements known as β -stabilizers (e.g., Fe, Cr, Mo, V, Nb, Ta, and W) contributes to stabilizing the β -phase at the expense of martensite [31,32,33,34]. Martensite displays a hexagonal close-packed (HCP) $\alpha^{\prime }$ or orthorhombic $\alpha^{\prime \prime }$ structure depending on the composition.

Previously, Lee et al. [22] developed a new ultrafine eutectic alloy, (Ti_70.5_Fe_29.5_)_99.0_Bi_1.0_, which possesses an excellent plasticity of up to 8.5% without a drastic reduction in the yield strength of 2100 MPa, applying the chemical heterogeneity characteristic among the constituent elements [22,35]. The detailed results of the microstructural investigation indicate that Bi tends to be saturated at the grain boundary of the lamellar matrix leading to the dissipation of the localized stresses. Furthermore, Song et al. [36] successfully achieved outstanding mechanical properties for the (Ti_63.5_Fe_30.5_)_94_Sn_6_ eutectic alloy by inducing structural heterogeneities [36,37,38,39,40]. It was found that the bimodal eutectic structure plays a critical role in hindering the propagation of deformation bands, which consequently leads to better ductility compared to the Ti Fe eutectic alloy. In addition, Lee et al. [22] suggested that the high strength and ductility of Ti–Fe–Sn ultrafine eutectic dendrite composites can be further achieved by the minute compositional changes based on the ternary phase diagram [25,26,41]. This compositional tuning enables the selection of primary dendrite phases in the β -Ti and Ti Fe lamellar matrix, such as β -Ti, TiFe, and Ti_3_Sn phases. The formation of intermetallic dendrites in the eutectic matrix could efficiently improve the yield strength rather than ductility due to their mechanical characteristics. In the case of Ti–Fe–Sn eutectic composites, however, the Ti_72_Fe_22_Sn_6_ alloy which consists of a solid-solution β -Ti primary dendrite, shows a comparable yield strength and high hardness compared to Ti Fe and Ti_3_Sn containing Ti–Fe–Sn eutectic alloys.

In this study, Ti–Fe–Sn alloys with β -Ti dendrites have been fabricated based on the Ti–Fe–Sn ternary phase diagram previously studied [25,26]. This particular alloy system is of interest because of its ability to form three different types of dendrites, which can be used to obtain preferred mechanical properties. The varying microstructural changes that can be made to obtain mechanical properties include the formation of a bimodal eutectic structure to enhance plasticity [4], changing the distribution of the FeTi phase to achieve a high fracture strength and plasticity [12], improving hardness with lamellae deformation and phase rearrangement [15], and enhancing the mechanical properties with dendrite phase selection [25], as well as the accumulation of slip bands in the β -Ti solid solution leading to work hardening [26] and morphology modulation to obtain better plasticity [27]. The changes in the microstructure listed are not exhaustive, and the mentioned examples only represent a subset of the potential modifications that can be implemented. Lamellar microstructures with needle-shaped primary dendrites were observed by secondary-electron microscopy (SEM) analysis in Ti_75_Fe_19_Sn_6_ and Ti_77_Fe_14.5_Sn_8.5_. The needle-shaped $\alpha^{\prime \prime }$ -Ti martensite (orthorhombic) phase within a β -Ti dendrite is observed by transmission-electron microscopy (TEM) analysis. The effect of a round-shaped β -Ti dendrite on improving macroscopic ductility without a trade-off between strength and plasticity is studied here.

## 2. Experimental Procedure

The Ti_72_Fe_22_Sn_6_, Ti_75_Fe_19_Sn_6_, and Ti_77_Fe_14.5_Sn_8.5_ alloys were prepared with Ti, Fe, and Sn elements of 99.99 weight percent (wt. %) purity by arc-melting casting under an argon atmosphere on a water-cooled Cu hearth. Before alloying, the arc melter chamber was evacuated to 10^−5^ torr, and Ti getter was used to consume traces of oxygen and hydrogen for all melting steps. To achieve a homogeneous distribution of elements, the ingot was melted more than 10 times, by flipping them over between each step. The samples were water-quenched after casting. The cooling rate during fabrication for all present alloys is 10–10^−2^ K/s. From these master alloys, rod samples with a 3 mm diameter and 50 mm length for mechanical tests were solidified in a Cu mold under an Ar atmosphere followed by direct casting into cylindrical rods using a suction casting facility. The phase identification of the alloys was performed by X-ray diffraction (XRD, D/MAX-2500/PC, RIGAKU, Seoul, Republic of Korea) with Cu-K α radiation. The microstructures of these alloys were examined by an SEM (JEOL JSM-6390, Seoul, Republic of Korea) equipped with an energy-dispersive spectrometer (EDS) and TEM (TECNAI-F20, Seoul, Republic of Korea). To investigate the in-depth microstructures by TEM, thin-foil samples were prepared by conventional ion milling (Gatan, model 600, Seoul, Republic of Korea). Cylindrical specimens with a 2:1 aspect ratio for compression tests were prepared. Mechanical properties were measured by uniaxial compression test at a strain rate of 10^−3^ s^−1^ at room temperature. The deformed and fractured samples were polished to observe the surface deformation morphology.

## 3. Results and Discussion

**Figure 1:**
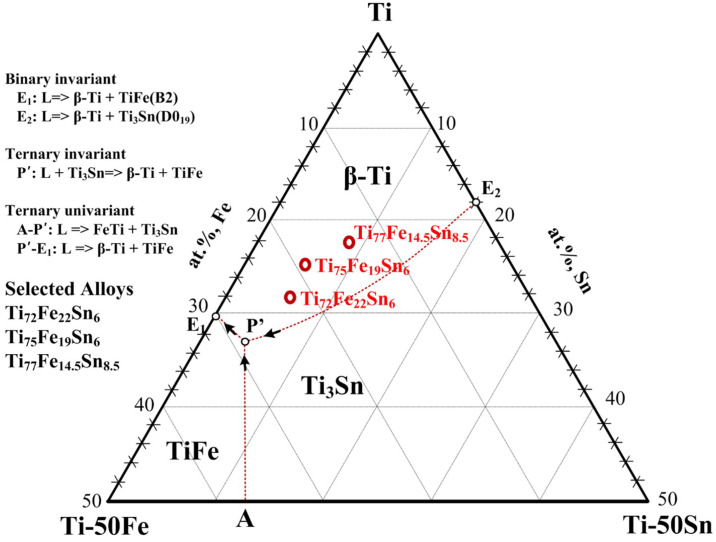
The ternary Ti–Fe–Sn phase diagram in the Ti-rich corner was investigated in this study. The dashed lines separate the liquid surface for β -Ti, TiFe, and Ti_3_Sn.

Figure 1 illustrates the Ti–Fe–Sn ternary phase diagram in the Ti-rich region, constructed using the previous experimental data [25,26,41]. The formation of the lamellar structure, comprising the β -Ti + TiFe, is feasible through ternary univariant (A-P′) and quasi-peritectic reactions (L+ Ti_3_Sn => β -Ti + TiFe). In this study, the focus was on eutectic–dendrite composites with compositions of Ti_72_Fe_22_Sn_6_, Ti_75_Fe_19_Sn_6_, and Ti_77_Fe_14.5_Sn_8.5_, which are highlighted as red dots in Figure 1. This particular region was chosen considering the inducement of β -Ti dendrites, thoroughly demonstrating their intrinsic characteristics and contribution to mechanical properties. As shown in Figure 1, there are three possible dendrite phases in the Ti–Fe–Sn alloy system, such as β -Ti, TiFe, and Ti_3_Sn. Among these primary dendrite phases, only β -Ti is a solid-solution, which is expected to have a better ductility than the other intermetallic compound phases (TiFe and Ti_3_Sn) [25]. It is further anticipated that, as the composition approaches the Ti-rich region within the Ti–Fe–Sn ternary phase diagram, the volume fraction of the eutectic matrix and β -Ti primary dendrites undergo gradual changes. A reduced yield strength is obtained with an increased volume fraction of the β -Ti dendrite. Thus, the composition closer to the β -Ti corner is favored. Hence, it is anticipated that a decrease in the volume fraction of the β -Ti dendrite should be accompanied by decreased plasticity. Martensite phase formation can be induced by controlling the dendrite morphology, which, in turn, is induced by undercooling in the experiments conducted herein. Based on the results, it can be concluded that dendrite morphology is a key factor in the enhancement of plasticity.

**Figure 2:**
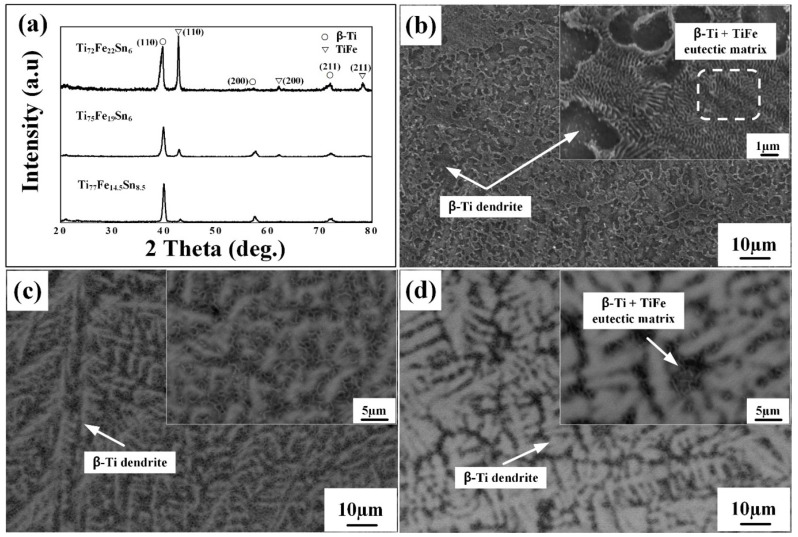
(**a**) XRD phase analysis and SEM micrographs of the Ti–Fe–Sn ultrafine eutectic composites; (**b**) SE image of Ti_72_Fe_22_Sn_6_ sample; (**c**) BSE image of Ti_75_Fe_19_Sn_6_; and (**d**) BSE image of Ti_77_Fe_14.5_Sn_8.5_.

Figure 2 shows the XRD patterns and the SEM micrographs for the Ti_72_Fe_22_Sn_6_, Ti_75_Fe_19_Sn_6_, and Ti_77_Fe_14.5_Sn_8.5_ alloys. The main diffraction peaks for all three alloys were identified as a combination of the BCC β -Ti (A2) solid solution and the TiFe (B2-type) intermetallic compound. As the alloy composition gradually shifts towards the β -Ti rich region, as illustrated in Figure 1, a notable decrease in the intensity of the TiFe phase peaks was observed, while the diffraction peaks corresponding to β -Ti show minimal variation. Figure 2b represents the secondary electron (SE) image of Ti_72_Fe_22_Sn_6_, whereas Figure 2c,d present the SEM–backscattered electron (BSE) images of Ti_75_Fe_19_Sn_6_ and Ti_77.5_Fe_14.5_Sn_8.5_ alloys. The Ti_72_Fe_22_Sn_6_alloy (Figure 2b) shows a typical ultrafine eutectic-dendrite composite microstructure, comprising a β -Ti + TiFe lamellar structured matrix with round-shaped β -Ti dendrites. The lamellar spacing of the eutectic matrix and the size of the round-shaped primary dendrite are about 300–500 nm and 5–10 μ m, respectively. The values were obtained using the software, ImageJ V1.8. The volume fraction of the micron-scale primary dendrite is estimated to be approximately 40.75%. The microstructures of Ti_75_Fe_19_Sn_6_ and Ti_77.5_Fe_14.5_Sn_8.5_ alloys (Figure 2c,d) indicate a slightly coarsened eutectic matrix and an increased volume fraction of primary β -Ti dendrite phase compared to the Ti_72_Fe_22_Sn_6_ alloy (Figure 2b). The lamellar spacing and approximate volume fraction of the primary dendrite for Ti_75_Fe_19_Sn_6_ and Ti_77_Fe_14.5_Sn_8.5_ alloys are about 600–900 nm, 700–1000 nm and 60.75%, 77.25%, respectively. The trends of the microstructure and volume fraction of the dendrite phase are closely aligned with those from the ternary phase diagram (Figure 1).

When the Ti content approaches 75 at. %, a noteworthy peak shift occurs not only in the volume fraction of dendrites but also in the morphology of the primary β -Ti dendrite. This trend causes a change in the dendrite structure from a round shape to a needle-like configuration. As the concentrations of Ti and Sn are further increased, this transformation intensifies, leading to a significant proliferation of needle-shaped β -Ti dendrites. Consequently, this substantial increase in needle-shaped dendrites correlates with a pronounced reduction in the TiFe peak intensity as observed in the XRD patterns (Figure 2a).

**Figure 3:**
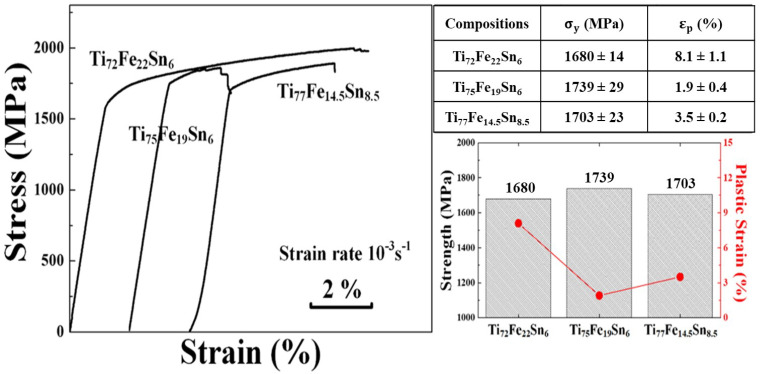
Engineering compressive SS curve of the Ti–Fe–Sn ultrafine eutectic composites at room temperature and obtained mechanical properties ( σ _y_, σ _f_, and ε _p_) of the Ti–Fe–Sn ultrafine eutectic composites. A table and graphical representation of the data has been added.

Figure 3 presents compressive engineering stress–strain (SS) curves for Ti–Fe–Sn ultrafine eutectic composites at room temperature. The Ti_72_Fe_22_Sn_6_ alloy, which consists of a round-shaped β -Ti primary dendrite, shows a yield strength ( σ _y_) of 1680 MPa; ultimate strength ( σ _f_) of 1979 MPa and the highest plastic strain ( ε _p_) of 8.1%, respectively. The yield strength and plastic strain values for Ti_75_Fe_19_Sn_6_ and Ti_77_Fe_14.5_Sn_8.5_ eutectic composites with a needle-shaped dendrite are 1739 MPa, 1703 MPa and 1.9%, 3.5%; respectively. The detailed values of mechanical properties in Ti–Fe–Sn eutectic alloys are summarized in Figure 3.

In contrast to previous observations, however, the present Ti_72_Fe_22_Sn_6_ alloy shows a competitive plastic strain with the Ti_75_Fe_19_Sn_6_ and Ti_77_Fe_14.5_Sn_8.5_ alloys, which possess substantial amounts of β -Ti dendrite volume fraction. These results imply that the degree of plasticity for the presented Ti–Fe–Sn alloy systems relies on not only the volume fraction of the primary dendrite but also the morphology of the dendrite. Hence, in-depth nano-scale structural investigations for Ti_72_Fe_22_Sn_6_ and Ti_77_Fe_14.5_Sn_8.5_ alloys were conducted by the TEM technique to distinguish the distinct characteristics of the β -Ti dendrite.

**Figure 4:**
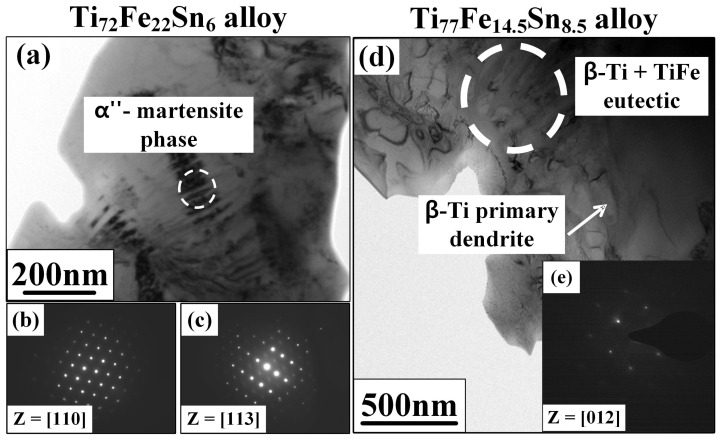
TEM bright-field (BF) image of: (**a**) Ti_72_Fe_22_ Sn_6_ eutectic composite and the selected-area electron diffraction (SAED) patterns; (**b**) [110] zone axis; (**c**) [113] zone axis of the β -Ti phase and secondary $\alpha^{\prime \prime }$ -martensite phase (one variant only); (**d**) Ti_77_Fe_14.5_Sn_8.5_ eutectic composite and the SAED patterns; and (**e**) [012] zone axis of the β -Ti phase.

Figure 4 presents the TEM-BF images and SAED patterns of Ti_72_Fe_22_Sn_6_ and Ti_77_Fe_14.5_Sn_8.5_ ultrafine eutectic composites. The TEM-BF image and SAED pattern obtained from Ti_77_Fe_14.5_Sn_8.5_ eutectic composite with the largest volume fraction of the β -Ti dendrite (Figure 4d,e) indicate the formation of a mixture of fine lamellar structures in a eutectic matrix and primary dendrite. The dendrite is identified as a β -Ti phase from the SAED pattern indexed with the [012] zone axes (Figure 4e). Figure 4a shows the TEM-BF image of the Ti_72_Fe_22_Sn_6_ eutectic composite with the lowest β -Ti dendrite volume fraction. Ti_77_Fe_14.5_Sn_8.5_ is known for a characteristic β -Ti dendrite phase; in contrast, nano-scaled needle-shaped phases embedded in primary β -Ti dendrite were observed in Ti_72_Fe_22_Sn_6_, as marked in Figure 4a. The chemical composition of the β -Ti dendrite phases is 76.86 at. % of Ti, 15.22 at. % of Fe, and 7.92 at. % of Sn, which was estimated by the energy-dispersive X-ray spectroscopy (EDX) analysis. Five spots were investigated using EDS and an average was taken. In general, Fe has been known as a β -Ti stabilizer and thus makes the β -Ti phase stable at low temperatures [22]. To further identify the distribution of the phases, the SAED patterns are obtained from the primary dendrite (Figure 4b) and the needle-shaped phase (Figure 4c) regions. The results of the SAED pattern are identified as a β -Ti solid solution with a [110] zone axis and the $\alpha^{\prime \prime }$ -Ti martensite phase with a [113] zone axis, indicating a mixture of β -Ti and $\alpha^{\prime \prime }$ -Ti phases in the primary solid-solution dendrite. These experimental observations suggest that, becoming closer toward quasi-peritectic reaction points (P’) from the Ti-rich corner in the Ti–Fe–Sn ternary phase diagram (Figure 1), the concentration of Fe and its phases in the β -Ti primary dendrite decreased with the increase in a lamellar structured matrix ( β -Ti +TiFe). Fe and its phases significantly contribute to the formation of the TiFe intermetallic compound layers in a matrix with the increase in TiFe diffraction intensity (Figure 2a). In highly alloyed Ti-alloy systems, which contain more than three elements, the structure of the $\alpha^{\prime \prime }$ -martensite becomes distorted, and the orthorhombic $\alpha^{\prime \prime }$ -martensite is formed during quenching. These trends of solidification result in the formation of a non-equilibrium $\alpha^{\prime \prime }$ -Ti martensitic phase in the Ti_72_Fe_22_Sn_6_ alloy [42]. Generally, the presence of an orthorhombic crystal structure, such as in $\alpha^{\prime \prime }$ -Ti, could improve the strength due to the higher stress requirement for plastic deformation with fewer slip systems than the BCC β -Ti phase, but it also leads to a reduction in ductility with stress localization along the $\alpha^{\prime \prime }$ -Ti phase [43,44]. However, the Ti_72_Fe_22_Sn_6_ alloy, which contained the $\alpha^{\prime \prime }$ -Ti martensitic phase, shows a similar yield strength with larger plasticity compared to the Ti_75_Fe_19_Sn_6_ and Ti_77_Fe_14.5_Sn_8.5_ alloys, as shown in the SS curves in Figure 3. These results could imply that the presence of small volume fractions of $\alpha^{\prime \prime }$ -martensitic phase could not efficiently improve the yield strength, but it facilitates controlling the dendrite morphology, as exhibited in the SEM images [Figure 2b–d]. It can be deduced that the nucleation of the $\alpha^{\prime \prime }$ -martensitic phase in the β -Ti dendrite hinders the directional growth of the primary dendrite, thus altering the morphology of the β -Ti dendrite to be rounded, and it plays a prominent role in the enhancement of ductility.

**Figure 5:**
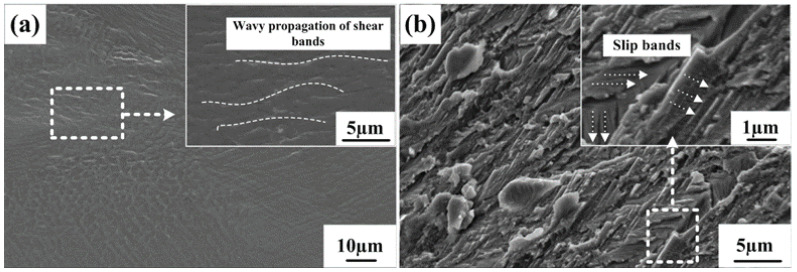
Secondary electron SEM images taken from the failed as-cast Ti_72_Fe_22_Sn_6_ sample: (**a**) lateral surface morphology; (**b**) fracture surface morphology.

In order to study the deformation behavior and investigate the effect of β -Ti dendrites on mechanical properties, the fracture and lateral surface examination were carried out in SEM, as shown in Figure 5. Figure 5a,b exhibit the SEM images obtained from the lateral and fracture surfaces of the failed Ti_72_Fe_22_Sn_6_ alloy, respectively. On the lateral surface, a striking feature is the abundance of deformation bands that traverse the specimen. This abundance of bands indicates a significant level of plastic strain experienced by the material before fracture. Notably, these deformation bands tend to align predominantly along the interfaces of dendrites, resulting in a remarkable and visually distinctive wavy pattern of shear bands. This phenomenon suggests that the dendrite interfaces contribute to accommodating the deformation and allowing the material to stretch and deform extensively before succumbing to fracture. Upon closer examination of the fracture surface of the Ti_72_Fe_22_Sn_6_ sample depicted in Figure 5b, a distinct set of intriguing features comes into view. Here, one can observe the presence of multiple stepped features with river-like patterns, indicative of the dynamic and complex fracture process that occurred. Furthermore, slip bands are observed to be homogeneously distributed within the dendrite phases, emphasizing the role of dendritic structures in accommodating deformation. This observation leads to an important inference: the round-shaped dendrites present in the Ti_72_Fe_22_Sn_6_ alloy seem to be highly effective in dispersing stress concentration during deformation. Needle-shaped dendrites are known to be the location of stress concentration leading to fracture and, hence, nodulization (round-shaped dendrite formation) is favored in ductile iron [45]. The spherical morphology of the ultrafine eutectic colony was effective in enhancing the plastic strain in the samples [27]. This dispersal of stress concentration helps achieve a large plastic strain in the alloy, setting it apart from other alloy systems characterized by needle-shaped dendrites.

Microstructural evolutions were observed as a function of β -Ti dendrite volume changes. In addition to that, we learned that the dendrite morphology is important for plasticity improvement and it can be controlled by inducing the formation of the α " phase in the β -Ti dendrite with an alloy composition change. Thus, compositional tuning enabled us to achieve better plasticity.

## 4. Summary

In this study, three different compositions of Ti–Fe–Sn ultrafine eutectic composites were selected, considering the distinct volume fractions and morphologies of β -Ti dendrite formed based on the Ti–Fe–Sn ternary phase diagram. The composites were investigated through fine compositional tuning at the Ti-rich corner. The conclusions of this study are described as follows:

1. The Ti_72_Fe_22_Sn_6_, Ti_75_Fe_19_Sn_6_, and Ti_77_Fe_14.5_Sn_8.5_ alloys consist of a β -Ti solid-solution phase and TiFe intermetallic compound without the presence of a Sn-related phase. The intensities of TiFe diffraction peaks gradually decreased with the increase in β -Ti primary dendrite volume fractions as the alloy compositions moved toward the Ti-rich corner.
2. Typical lamellar microstructures with needle-shaped primary dendrites for Ti_75_Fe_19_Sn_6_ and Ti_77_Fe_14.5_Sn_8.5_ alloys are observed by SEM analysis. The increase in the β -Ti primary dendrite results in improved ductility due to the better accumulation of dislocations. The Ti_72_Fe_22_Sn_6_ alloy, however, exhibits a much higher ε _p_ of 8.1% than other alloys, even though it contained the smallest volume fraction of the β -Ti primary dendrite. Enhanced mechanical properties are achieved when the propagation of shear bands and cracks is restricted. Thus, the pile-up or accumulation of dislocations leads to improved plasticity and a work-hardening response [3,17]. Dislocation pile-up can lead to strain hardening which leads to improved plasticity. The pile-up also enables the homogeneous distribution of deformation across the material. This prevents localized stress concentration and promotes a uniform plastic response.
3. It is found that the β -Ti dendrites in the Ti_72_Fe_22_Sn_6_ alloy are composed of a lower Fe content, which is well known as a β -Ti stabilizer. These chemical gradients during solidification could induce the under-cooling effects. Hence, the needle-shaped $\alpha^{\prime \prime }$ -Ti martensite (orthorhombic) phase within the β -Ti dendrite is observed by the TEM analysis. Since martensite crystallizes the earliest in the matrix, it hinders dendritic growth. These features of the pre-formation of the martensitic phase facilitate the formation of round-shape dendrites.
4. The wavy propagation of abundant deformation bands and multiple steps on the fracture surface can cause ductile deformation. Furthermore, the formation of a large number of slip bands plays an important role in plasticity during deformation.

These overall results suggest that the morphology and volume fractions of primary dendrite can be efficiently controlled by minor compositional tuning. The formation of the round-shaped β -Ti dendrite, which is induced by the presence of the $\alpha^{\prime \prime }$ -Ti martensitic phase, effectively improved the macroscopic ductility without a trade-off between strength and plasticity.

## Data Availability

Data are contained within the article.
